# Blunted Activation of Rho-Kinase in Yak Pulmonary Circulation

**DOI:** 10.1155/2015/720250

**Published:** 2015-01-14

**Authors:** Takeshi Ishizaki, Shiro Mizuno, Akio Sakai, Shigeru Matsukawa, Baktybek Kojonazarov, Baiserkeev Zamirbek, Yukihiro Umeda, Miwa Morikawa, Masaki Anzai, Tamotsu Ishizuka, Almaz Aldashev

**Affiliations:** ^1^Department of Fundamental Nursing and Medicine, University of Fukui, Fukui 910-1193, Japan; ^2^Department of Respiratory Medicine, Kanazawa Medical University, Ishikawa 920-0265, Japan; ^3^Department of Health Science, Matsumoto University, Matsumoto 390-1295, Japan; ^4^Life Science Research Laboratory, University of Fukui, Fukui 910-1193, Japan; ^5^Laboratory of Molecular and Cell Biology, Institute of Molecular Biology and Medicine, 720040 Bishkek, Kyrgyzstan; ^6^Excellence Cluster Cardio-Pulmonary System, Universities of Giessen & Marburg Lung Center, 35392 Giessen, Germany; ^7^Department of General Surgery, Kyrgyz State Medical Academy, 720020 Bishkek, Kyrgyzstan; ^8^Department of Internal Medicine, University of Fukui, Fukui 910-1193, Japan

## Abstract

Yaks have adapted to high altitude and they do not develop hypoxic pulmonary hypertension. Although we previously identified the important role of augmented nitric oxide synthase activity in the yak pulmonary circulatory system, evidence of the direct involvement of Rho-kinase as a basal vascular tone regulator is lacking. Four domesticated male pure-bred yaks and four bulls that were born and raised at an altitude of 3000 m in the Tien-Shan mountains were studied at an altitude of 3,100 m. Mean pulmonary artery pressure (mPAP) was measured before and after fasudil (60 mg in 20 mL of saline) was intravenously administered using a Swan-Ganz catheter at a rate of 3.3 mL/min for 30 min. Fasudil decreased mPAP in bulls from 67.8±14.9 to 32.3±5.3 mmHg (*P* < 0.05) after 15 min and the level was maintained for 30 min, but it merely blunted mPAP in yaks from 28.2±4.5 to 25.1±11.1 and 23.2±2.7 mmHg after 5 and 30 min, respectively. These findings comprise the first evidence of a modest role of Rho-kinase in the maintenance of pulmonary artery pressure in the yak.

## 1. Introduction

Hypoxic pulmonary hypertension (HPH) is a common complication of cardiopulmonary diseases among immigrating humans and other animals that live at high altitudes [1–5]. It is characterized by pulmonary vascular remodeling and right ventricular dysfunction as well as pulmonary arterial hypertension. However, despite living under hypoxic circumstances, animals such as llama, yak, pika, and Tibetan sheep have low pulmonary vascular tone, attenuated hypoxic pulmonary vasoconstriction, and normal right/left ventricle weight [6–11]. Thus, such adaptations allow them to be active at high altitudes. In terms of the biophysiological mechanisms of the intact yak pulmonary vascular system, we previously reported that nitric oxide (NO) production is augmented in yak pulmonary circulation [11]. We also found elevated activity of dimethylarginine dimethylaminohydrolase (DDAH), which suppresses asymmetric dimethylarginine, an endogenous inhibitor of nitric oxide synthase (NOS) [12] in yak lung tissue (unpublished observation). Rho-kinase plays an essential role in regulating vascular smooth muscle (VSM) contraction, VSM cell proliferation, and migration [13], and sustained Rho-kinase mediated vasoconstriction seems to be closely associated with chronic hypoxia-induced pulmonary hypertension [14–16]. Furthermore, accumulating evidence suggests an inverse relationship between endothelial nitric oxide synthase (eNOS) and small GTPase RhoA/Rho-kinase since small GTPase RhoA/Rho-kinase negatively regulates eNOS phosphorylation through the inhibition of protein kinase B/Act in human endothelial cells [17]. Rho-kinase might negatively regulate eNOS expression and activation, as well as NO bioavailability [18, 19], and it mediates the hypoxia-induced downregulation of endothelial NO synthase [20]. Therefore, we considered that basal Rho-kinase activity functions at a basal level in the yak pulmonary vasculature. We thus assessed pulmonary arterial pressure using a Rho-kinase inhibitor (PAP) in yaks and bulls as controls in their natural habitat in the Tien-Shan mountains.

## 2. Materials and Methods

### 2.1. Animals

We used male domesticated pure-bred yaks (*n* = 4; age, 2 y; weight, 200 kg) and bulls (*n* = 4; age, 2; weight, 200 kg) that were born and raised at altitudes of 3000~3400 and 3200~3400 m in Archaly and Taragay, Kyrgyzstan, respectively. Experiments proceeded at altitudes of 3000 and 3100 m in Archaly and Taragay, respectively.

The Ethics Committee of the National Center of Cardiology and Internal Medicine (Bishkek, Kyrgyzstan) approved this study.

### 2.2. Chemicals

Yaks and bulls were intravenously injected with the Rho-kinase inhibitor fasudil hydrochloride hydrate (Eril; Asahi Kasei Pharma Corporation, Tokyo, Japan), which is clinically applied in Japan to prevent cerebral artery vasospasm (subarachnoid hemorrhage). Its pharmacodynamic half-life is 16 min when injected into rat vessels. Fasudil hinders the activity of Rho-kinase and myosin light chain kinase with Ki values of 0.35 and 36 *μ*M, respectively, in vitro.

### 2.3. Hemodynamic Studies

A balloon-tipped pulmonary arterial catheter was inserted percutaneously into the right internal jugular vein and then advanced to the pulmonary artery to measure pulmonary artery pressure under general anesthesia [11]. All studies started after the animals had fully recovered from the anesthesia. Correct placement of the catheters was monitored using an NR-2000 pressure wave tracer (Keyence Corp., Osaka, Japan) and PAP was recorded. Mean PAP (mPAP) was measured at 0, 5, 10, 15, 20, and 30 min after the intravenous administration of fasudil (60 mg in 20 mL saline; flow rate, 3.3 mL/min).

### 2.4. Histopathological Analysis

After completing the hemodynamic studies, the heart and lungs were removed en bloc from some animals and then the ratios of right to left ventricular (RV/LV) weight were measured. Fixed lung tissues were histopathologically analyzed.

### 2.5. Statistical Analysis

All values are expressed as means ± standard error. Data were statistically analyzed using one-way analysis of variance (ANOVA) followed by the Bonferroni *t*-test. Responses to fasudil in each group of animals were assessed using a paired *t*-test. *P* < 0.05 was considered to represent a statistically significant difference.

## 3. Results

### 3.1. Effects of Fasudil Infusion on Pulmonary Hemodynamics

**Figure 1:**
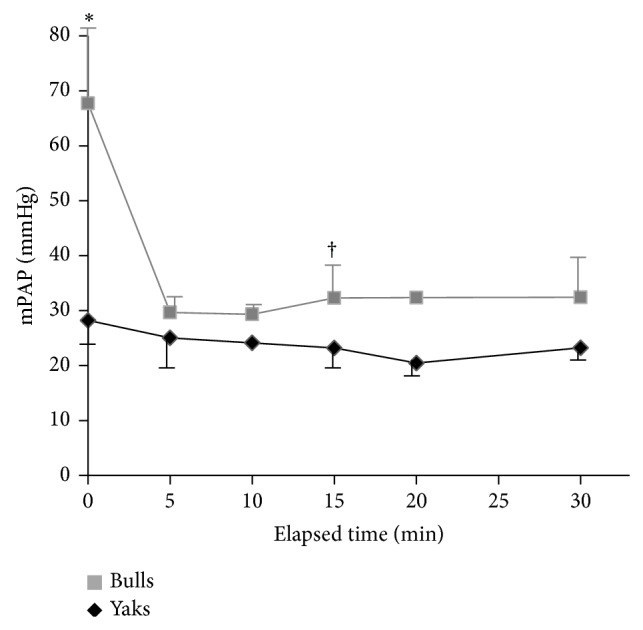
Sequential changes of mPAP in yaks and bulls after fasudil administration. Squares and diamonds represent 3-4 animals except at 5 and 15 min (*n* = 2 yaks). Bars indicate SEM. ^*^Significant difference between groups (*P* < 0.05). ^†^ *P* < 0.05 versus baseline value for bulls.

**Figure 2:**
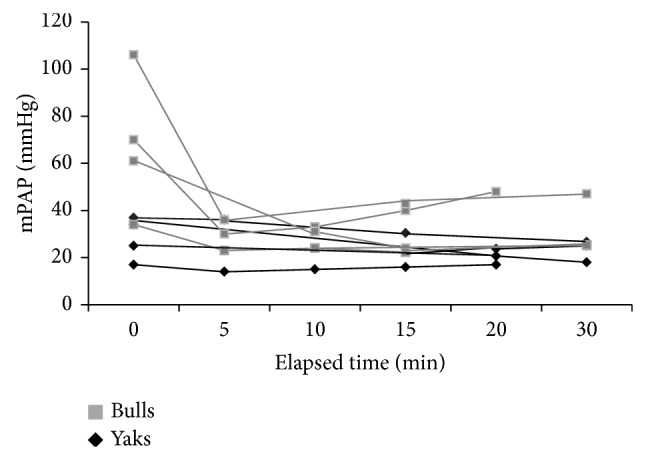
Sequential changes of mPAP in individual yaks and bulls after fasudil administration.

The basal mPAP of bulls and yaks significantly differed (67.7 versus 28.2 mmHg, *P* < 0.05; Figure 1). Fasudil caused an immediate decrease in the mPAP of bulls to 40% compared with the basal value and maintained that level for at least 30 min (*P* < 0.05 at 15 min versus basal value). In contrast, the mPAP of yaks weakly responded to fasudil and the difference did not reach significance (Figure 1). The mPAP reaction to fasudil was identical in all yaks (Figure 2), whereas one bull had low vascular tone compared with the other three.

### 3.2. Histopathological Analysis

**Figure 3:**
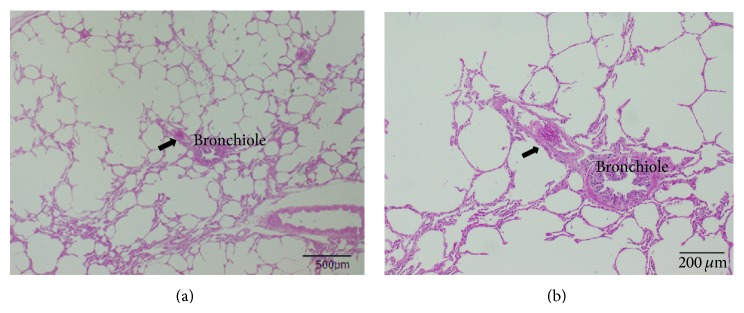
Representative photomicrograph of small pulmonary artery from pure-bred, domesticated two-year-old male yak living at high altitude. Views of lung tissue at low (a) and high (b) magnification show absent thickening in pulmonary artery with accompanying small airways (H&E stain). The RV/LV and mPAP of this yak were 0.315 and 25 mmHg, respectively. Arrow: small pulmonary artery.

**Figure 4:**
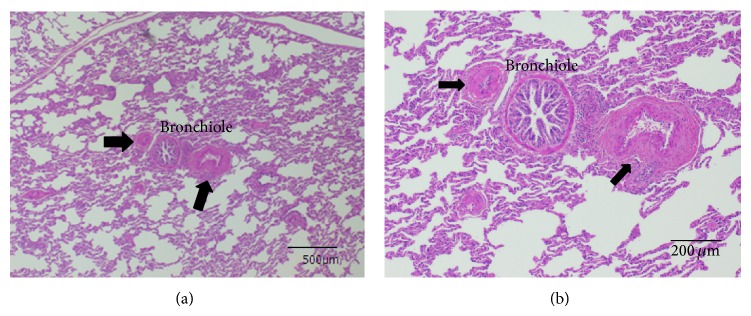
Representative photomicrograph of small pulmonary artery from two-year-old male bull born and raised at altitude of 3000 m. Views of lung tissue at low (a) and high (b) magnification show prominent medial thickness and enlarged outer layer of pulmonary artery with accompanying small airway (H&E stain). Ratio of RV/LV and mPAP of this bull were 0.545 and 70 mmHg, respectively. Arrows: small pulmonary artery.

The small pulmonary arteries of yaks did not have thickened intimal and medial walls or a thickened adventitial layer, compared with those of the bulls (Figures 3 and 4), in which medial thickness was prominent and the outer layer was enlarged.

## 4. Discussion

The notable finding of the present study is that Rho-kinase activity is not augmented in yak pulmonary arteries compared with those of bulls, suggesting a reason for the relatively low pulmonary vascular tone of yaks despite living in hypoxic circumstances. Zhou and Liao [21] noted that some basal activity is probably required to maintain vascular homeostasis, and thus fasudil might scarcely affect basal Rho-kinase activity in the yak pulmonary vasculature. To our knowledge, this is the first study to suggest that Rho-kinase activation is blunted in yak pulmonary vessels. Since many investigators have noted Rho-kinase-induced eNOS suppression [17–20], blunted Rho-kinase activation might result in intact/augmented eNOS activation in yak pulmonary vasculature, which we previously described [11]. Conversely, intact/augmented eNOS activation of the yak pulmonary circulation might control Rho-kinase activity [22, 23]. Rho-kinase activation is involved in hypoxia-induced vasoconstriction or hypoxia-induced pulmonary hypertension in animal models [14, 24–26]. We also noted that the Rho-kinase inhibitor, fasudil, decreases systolic PAP in humans who live at high altitudes in Kyrgyzstan [27]. That study was the first to directly show that fasudil could decrease PAP even in humans with hypoxic (high-altitude) pulmonary hypertension. Thus, accumulating evidence supports the notion that Rho-kinase activation plays a central role in sustaining pulmonary vascular constriction in both the acute and chronic phases of hypoxic pulmonary hypertension. We showed here that fasudil decreased mPAP in bulls that have elevated mPAP, thus supporting the involvement of Rho-kinase activation in HPH. Since brisket disease in cattle [2, 28] is taken as an animal model of hypoxic pulmonary hypertension at high altitude, the molecular pathway of pulmonary hypertension in cattle should be investigated by focusing on Rho-kinase activity and/or related gene expression.

The Rho-kinase inhibitor, fasudil, can reverse vasoconstriction induced by ET-1 [29] and serotonin [30] and attenuate angiotensin II- (AII-) induced cardiac hypertrophy [31] and endothelin- (ET-) induced cardiac myocyte hypertrophy [32], and the potent vasoconstrictors including AII [33], ET [29], and serotonin [34] can activate the Rho/Rho-kinase pathway. Thus, fasudil might exert broader and more potent Rho-kinase inhibition and other actions upon the pulmonary vascular tone of bulls. Although we did not compare the effect of inhibitors of the above vasoconstrictors, we believe that the most likely explanation for the fasudil-induced acute decrease in pulmonary artery pressure in chronically hypoxic bulls is caused by the inhibition of vascular constriction caused by Rho-kinase activation [35].

In terms of yak PAP, Anand et al. reported that it ranges from 18 to 21 mmHg in elderly (age, 8–13 y) males [7] and Ishizaki et al. noted mPAP of 34 mmHg in 5–7-month-old male yaks exposed to 11–13% O^2^ [11]. Thus, although the ages of the yaks differed between these and the present study and the zero reference point probably also differed, the mPAP of 28.4 mmHg in the present study seems comparable. However, one of the four bulls weakly responded to fasudil. Anand et al. [7] studied stols (cross between dzo and bulls that resemble cows) at an altitude of about 4500 m in Ladakh, India. Half of those examined had PAP values and resistance similar to those of high-altitude yaks. Regardless, we cannot precisely explain the extraordinarily hyporeactive pulmonary vascular tone of one of the four bulls under sustained hypoxia in the present study. The yaks and bulls studied herein lived at the same altitude in the same area and grazed at the same pastures, and none were fed hay. Perhaps the circumstances had similar effects on the two species.

Our histopathological findings were in accordance with those of Durmowicz et al. [9] who noted that yaks have thinner-walled pulmonary vessels than calves that had lived at an altitude of 3,000 m. Calves that died of brisket disease had small pulmonary arteries (100–300 *μ*m), increased medial thickness, elastin deposition, and a distinct adventitial layer. These features were similar to those in the lungs of the bulls in the present study.

Several limitations are associated with the present study. Because the altitude at the experimental site imposed technical difficulties, the activity and/or localization of Rho-kinase could not be directly measured in the animal lungs. We did not compare the effects of fasudil using inhibitors of the potent vasoconstrictors, AII and ET. However, to collect animals and remain at high altitudes for long was beyond our capacity. We selected four bulls, one of which did not have elevated pulmonary vascular tone. Genomic identification of test animals would be preferable for future similar studies. Some sequential mPAP data from both species were lost. Regardless, we aimed to measure mPAP at all scheduled time points.

## 5. Conclusions

The pulmonary circulation of the yak is resistant to the effects of high-altitude hypoxia and to the effects of fasudil. Whether or not Rho-kinase plays a role in maintaining low pulmonary artery pressure in altitude-adapted yaks could not be defined in the present study. However, we believe that the Rho-kinase activation that is involved in hypoxia-induced pulmonary hypertension would not profoundly affect yak pulmonary vasculature. The present results might provide the basis for future genomic investigations that could result in the development of novel strategies for treating pulmonary hypertension.

## References

[1] Grover R. F. (1965). Pulmonary circulation in animals and man at high altitude. *Annals of the New York Academy of Sciences*.

[2] Hecht H. H., Kuida H., Lange R. L., Thorne J. L., Brown A. M. (1962). Brisket disease. II. Clinical features and hemodynamic observations in altitude-dependent right heart failure of cattle. *The American Journal of Medicine*.

[3] Tucker A., McMurtry I. F., Reeves J. T., Alexander A. F., Will D. H., Grover R. F. (1975). Lung vascular smooth muscle as a determinant of pulmonary hypertension at high altitude. *American Journal of Physiology*.

[4] Maggiorini M., Léon-Velarde F. (2003). High-altitude pulmonary hypertension: a pathophysiological entity to different diseases. *European Respiratory Journal*.

[5] Ghofrani H. A., Voswinckel R., Reichenberger F. (2006). Hypoxia- and non-hypoxia-related pulmonary hypertension—established and new therapies. *Cardiovascular Research*.

[6] Harris P., Heath D., Smith P. (1982). Pulmonary circulation of llama at high and low altitudes. *Thorax*.

[7] Anand I. S., Harris E., Ferrari R., Pearce P. (1986). Pulmonary haemodynamics of the yak, cattle, and cross breeds at high altitude. *Thorax*.

[8] Sakai A., Ueda G., Yanagidaira Y., Takeoka M., Tang G., Zang Y., Ueda G., Voelkel N. F. (1988). Physiological characteristics of pika, ochotona, high-altitude adapted animals. *High-Altitude Medical Science*.

[9] Durmowicz A. G., Hofmeister S., Kadyraliev T. K., Aldashev A. A., Stenmark K. R. (1993). Functional and structural adaptation of the yak pulmonary circulation to residence at high altitude. *Journal of Applied Physiology*.

[10] Koizumi T., Ruan Z., Sakai A. (2004). Contribution of nitric oxide to adaptation of tibetan sheep to high altitude. *Respiratory Physiology & Neurobiology*.

[11] Ishizaki T., Koizumi T., Ruan Z., Wang Z., Chen Q., Sakai A. (2005). Nitric oxide inhibitor altitude-dependently elevates pulmonary arterial pressure in high-altitude adapted yaks. *Respiratory Physiology and Neurobiology*.

[12] Pullamsetti S., Kiss L., Ghofrani H. A. (2005). Increased levels and reduced catabolism of asymmetric and symmetric dimethylarginines in pulmonary hypertension. *FASEB Journal*.

[13] Sauzeau V., Le Mellionnec E., Bertoglio J., Scalbert E., Pacaud P., Loirand G. (2001). Human urotensin II-induced contraction and arterial smooth muscle cell proliferation are mediated by RhoA and Rho-kinase. *Circulation Research*.

[14] Wang Z., Jin N., Ganguli S., Swartz D. R., Li L., Rhoades R. A. (2001). Rho-kinase activation is involved in hypoxia-induced pulmonary vasoconstriction. *American Journal of Respiratory Cell and Molecular Biology*.

[15] Nagaoka T., Morio Y., Casanova N. (2004). Rho/Rho kinase signaling mediates increased basal pulmonary vascular tone in chronically hypoxic rats. *American Journal of Physiology—Lung Cellular and Molecular Physiology*.

[16] Do.e Z., Fukumoto Y., Takaki A. (2009). Evidence for Rho-kinase activation in patients with pulmonary arterial hypertension. *Circulation Journal*.

[17] Ming X.-F., Viswambharan H., Barandier C. (2002). Rho GTPase/Rho kinase negatively regulates endothelial nitric oxide synthase phosphorylation through the inhibition of protein kinase B/Akt in human endothelial cells. *Molecular and Cellular Biology*.

[18] Laufs U., Liao J. K. (1998). Post-transcriptional regulation of endothelial nitric oxide synthase mRNA stability by Rho GTPase. *The Journal of Biological Chemistry*.

[19] Rikitake Y., Liao J. K. (2005). Rho GTPases, statins, and nitric oxide. *Circulation Research*.

[20] Takemoto M., Sun J., Hiroki J., Shimokawa H., Liao J. K. (2002). Rho-kinase mediates hypoxia-induced downregulation of endothelial nitric oxide synthase. *Circulation*.

[21] Zhou Q., Liao J. K. (2009). Rho kinase: an important mediator of atherosclerosis and vascular disease. *Current Pharmaceutical Design*.

[22] Sauzeau V., Le Jeune H., Cario-Toumaniantz C. (2000). Cyclic GMP-dependent protein kinase signaling pathway inhibits RhoA-induced Ca^2+^ sensitization of contraction in vascular smooth muscle. *The Journal of Biological Chemistry*.

[23] Zuckerbraun B. S., Stoyanovsky D. A., Sengupta R. (2007). Nitric oxide-induced inhibition of smooth muscle cell proliferation involves S-nitrosation and inactivation of RhoA. *The American Journal of Physiology—Cell Physiology*.

[24] Robertson T. P., Dipp M., Ward J. P. T., Aaronson P. I., Evans A. M. (2000). Inhibition of sustained hypoxic vasoconstriction by Y-27632 in isolated intrapulmonary arteries and perfused lung of the rat. *British Journal of Pharmacology*.

[25] Fagan K. A., Oka M., Bauer N. R. (2004). Attenuation of acute hypoxic pulmonary vasoconstriction and hypoxic pulmonary hypertension in mice by inhibition of Rho-kinase. *American Journal of Physiology: Lung Cellular and Molecular Physiology*.

[26] Hyvelin J.-M., Howell K., Nichol A., Costello C. M., Preston R. J., McLoughlin P. (2005). Inhibition of Rho-kinase attenuates hypoxia-induced angiogenesis in the pulmonary circulation. *Circulation Research*.

[27] Kojonazarov B., Myrzaakhmatova A., Sooronbaev T., Ishizaki T., Aldashev A. (2012). Effects of fasudil in patients with high-altitude pulmonary hypertension. *European Respiratory Journal*.

[28] Newman J. H., Holt T. N., Hedges L. K. (2011). High-altitude pulmonary hypertension in cattle (brisket disease): candidate genes and gene expression profiling of peripheral blood mononuclear cells. *Pulmonary Circulation*.

[29] Weigand L., Sylvester J. T., Shimoda L. A. (2006). Mechanisms of endothelin-1-induced contraction in pulmonary arteries from chronically hypoxic rats. *American Journal of Physiology: Lung Cellular and Molecular Physiology*.

[30] Sakurada S., Okamoto H., Takuwa N., Sugimoto N., Takuwa Y. (2001). Rho activation in excitatory agonist-stimulated vascular smooth muscle. *American Journal of Physiology: Cell Physiology*.

[31] Wang Y. X., da Cunha V., Martin-Mcnulty B. (2005). Inhibition of Rho-kinase by fasudil attenuated angiotensin II-induced cardiac hypertrophy in apolipoprotein e deficient mice. *European Journal of Pharmacology*.

[32] Satoh S.-I., Kawasaki K., Ikegaki I., Asano T., Shimokawa H. (2012). Evidence of a direct cellular protective effect of Rho-kinase inhibitors on endothelin-induced cardiac myocyte hypertrophy. *Biochemical and Biophysical Research Communications*.

[33] Takeda K., Ichiki T., Tokunou T. (2001). Critical role of rho-kinase and MEK/EFK pathways for angiotensin II-induced plasminogen activator inhibitor type-1 gene expression. *Arteriosclerosis, Thrombosis, and Vascular Biology*.

[34] Guilluy C., Eddahibi S., Agard C. (2009). RhoA and Rho kinase activation in human pulmonary hypertension: role of 5-HT signaling. *The American Journal of Respiratory and Critical Care Medicine*.

[35] Oka M., Homma N., Taraseviciene-Stewart L. (2007). Rho kinase-mediated vasoconstriction is important in severe occlusive pulmonary arterial hypertension in rats. *Circulation Research*.

